# Visual Acuity alterations in heavily impaired Congenital Zika Syndrome (CZS) children

**DOI:** 10.3389/fopht.2022.948409

**Published:** 2022-11-29

**Authors:** Luiz C. P. Baran, Diego da S. Lima, Leonardo A. Silva, Heydi S. Tabares, Sarah L. Dias, Andrea Araújo Zin, Maria E. L. Moreira, Marcelo F. da Costa, Dora F. Ventura

**Affiliations:** ^1^ Department of Experimental Psychology, Institute of Psychology, University of São Paulo, São Paulo, SP, Brazil; ^2^ Nucleus of Neurosciences and Behavior, University of São Paulo, São Paulo, SP, Brazil; ^3^ Clinical Research Unit, National Institute of Women, Children and Teenagers Fernandes Figueira, Oswaldo Cruz Foundation, Rio de Janeiro, Brazil

**Keywords:** Zika, ZIKV, virus, visual acuity, VA

## Abstract

**Introduction:**

This study aimed to assess visual acuity (VA) in Congenital Zika Syndrome (CZS)-children to evaluate visual loss. To that end we evaluated 41 CZS - children, from Rio de Janeiro using Teller Acuity Cards.

**Methods:**

To asses VA, we evaluated 41 CZS - children, from Rio de Janeiro using Teller Acuity Cards. The children had Zika virus-infection confirmed by reverse transcription–polymerase chain reaction (RT-PCR) or clinical evaluation.

**Results:**

In 39 out of 41 (95%) children, the VA scores were below normative values, while in 10 cases, VA was only marginally below normal; in the remaining 29 cases, VA was more than 0.15 logMAR below the lower limit. There was no correlation between VA and the cognitive domain tasks, although there was a correlation between VA and motor domain tasks. Thirty-seven children performed at least one task in the cognitive set, while fourteen children did not perform any task in the motor set. Children with VA above the lower limit performed better in the cognitive and motor tasks.

**Discussion:**

We concluded that ZIKV- infected children with CZS were highly VA impaired which correlated with motor performance, but not with cognitive performance. Part of the children had VA within the normal limits and displayed better performance in the cognitive and motor sets. Therefore, even if heavily impaired, most children had some degree of VA and visual function.

## Introduction

Gestational Zika virus (ZIKV) infection may lead to congenital Zika syndrome (CZS) (1–4), with microcephaly as its most known manifestation. In Brazil, where the pandemic had a high impact, a great number of cases of microcephaly were reported in 2015–2016 (5). There was a clear asymmetry in the regional occurrence of CZS, which was more frequent in the northeast region of Brazil and in Rio de Janeiro and Cuiabá. A causal link between ZIKV infection and the occurrence of microcephaly was inferred by a temporal correlation and was later experimentally confirmed (1, 6, 7). CZS is not restricted to microcephaly and has a myriad of manifestations, due chiefly to neurological impairment and massive intracranial volume loss (1). The features of the CZS spectrum include a partially collapsed cranium; neurological effects such as thin cerebral cortices, seizures, polymicrogyria, and subcortical calcifications; an increase in cerebral fluid spaces (ventriculomegaly); chiasmal atrophy; hypoplasia or loss of the corpus callosum; decreased myelination; cerebellar hypoplasia; and brainstem and basal ganglia calcifications, along with somatic abnormalities such as hypertonia, limb contracture, arthrogryposis (joint stiffening), altered craniofacial proportions, spasms, irritability, problems in swallowing, and hearing loss (1, 3, 4, 8).

CZS also affects the visual system. Its clinical manifestations include chorioretinal atrophy, macular pigmentary mottling, vascular changes, retinal focal spots, optic nerve anomalies, optic nerve atrophy, microphthalmia, iris coloboma, cataracts, and intraocular calcifications (1, 2, 9–11). Infection of the central nervous system might result in occipital volume loss, as well as eye motility issues such as strabismus, nystagmus, and accommodative capacity impairment (8, 12).

Although some studies focusing on visual and ocular alterations due to ZIKV infection have examined the impact of these vision-threatening events since the beginning of the recent ZIKV epidemic (2, 9, 11–18), the full scope of the effects of CZS on the visual system has not been completely characterized. In a cohort in the northeast of Brazil, where the highest rate of children born with CZS during the 2015–2016 ZIKV epidemic was seen, Ventura et al. (2) reported visual acuity (VA) losses in 76% of children with CZS (*n* = 25, all with microcephaly), failure to detect a low-contrast pattern in 65% (*n* = 19), and failure to achieve at least one visual development milestone in 97% (*n* = 29) of the examined children, apart from eye movement conditions. Ventura et al. (13) found VA deficits in approximately 85% of a larger sample (*N* = 119). These studies were carried out in populations from Northeast Brazil. More recently, Henderson et al. (8), in another study conducted in Northeast Brazil, in the state of Pernambuco, evaluated 70 children with ZIKV infection and, correlating the neuroimaging findings with visual and ocular assessments, found that all of the children with VA data available showed VA deficits; furthermore, of the VA-impaired children without eye anomalies, all had visual pathway abnormalities on neuroimaging, with100% of these infants having occipital cortical volume loss.

The impact of CZS on VA was also assessed by our team in a cohort from southeastern Brazil (Jundiaí, São Paulo), a region that presented a different epidemiological profile: although the ZIKV infection rates in the general population were high, the incidence of CZS was relatively low (19). Baran et al. (17) found that babies exposed to maternal ZIKV infection during pregnancy but did not become infected showed VA scores within the normal limit. Conversely, in the group of babies who acquired infection during gestation (*n* = 24), 5 (21%) had VA impairment, two of whom with microcephaly. Moreover, when examined as a group, the infected children showed a slower VA development rate compared with those in the control and exposed groups (17, 18).

Visual deficits were also observed in a sample from the state of Rio de Janeiro (11, 15, 16), the region with the highest incidence of CZS outside of northeastern Brazil (19). In this sample, 30% of the patients (*N* = 173) did not meet the requirements of a visual screening test that assessed the child’s capacity to fixate on a single monochromatic pattern and follow it with the gaze (the fix-and-follow test). This examination, while useful for identifying children with severe VA deficits, does not yield a threshold estimate, which is required for a direct comparison with previous studies. Moreover, since the pattern used in the test had low spatial frequency, mild VA losses might have gone undetected.

In the present study, we measured the VA in infants and children who had been exposed to ZIKV during gestation and who then developed CZS. Our aim was to better characterize the incidence and magnitude of vision loss in patients with CZS by assessing VA, a quantitative and universally measured indicator of visual function. VA was assessed behaviorally using a clinical version of the Teller Acuity Cards (TAC) (20–25). The TAC procedure is well established as an efficient and reliable instrument to measure VA in young children in a clinical setting (21–24).

By examining a population from another region, selected using different inclusion criteria, in a cohort of older, more impaired children, we further characterized the spread of the virus in Brazil, aiming to continue to help document the diversity of the impact of ZIKV in different regions of the country. These differences may be due to a variety of factors, including strain differences, host susceptibility (26), water contamination (27), or malnutrition (28). Additionally, it is critical to document and characterize the visual function alterations in CZS children with and without microcephaly.

## Materials and methods

This research is in line with the tenets of the Declaration of Helsinki (29) and was approved by the Ethics Committee for Human Research of the University of São Paulo’s Institute of Psychology (no. 67031216.0.0000.5561) and by the Ethics Committee for Human Research of the Instituto Fernandes Figueira (IFF)—Fiocruz (no. 526756616000005269). An informed consent form was signed by the parent or accompanying adult of the child after an explanation of the nature and purpose of the study was given.

The children examined were part of the Vertical Exposure to Zika Virus and Its Consequences for Child Neurodevelopment cohort, registered under NCT03255369 at the NIH Clinical Trials database (11, 15, 16). Methodological details of the enrollment criteria for patients can be found on the Clinical Trials page for the cohort (https://clinicaltrials.gov/ct2/show/NCT03255369).

The cohort was assembled and followed clinically in the IFF Fiocruz by the research team of the Institute under the guidance of co-author AZ. For the present study, we recruited children born from mothers with suspected ZIKV infection during pregnancy (who showed symptoms such as rash, arthralgia, myalgia, and fever) and who fulfilled one or more of the following criteria: a) positive reverse transcription polymerase chain reaction (RT-PCR) sample for ZIKV, from either pregnant mother or from the child within 10 days after birth; b) presence of structural congenital alterations detected *via* ultrasound; and c) clinical manifestations typical of CZS (such as microcephaly and eye alterations) detected *via* clinical examination after birth. The quantitative RT-PCR (RT-qPCR), serological test, and clinical examination (including fundoscopic evaluation) for ZIKV infection were performed by the research team at IFF Fiocruz (11, 15, 16). Pregnant women with chromosomal abnormalities detected during fetal life or childbirth were excluded (11, 15, 16).

The TAC test consists of 15 gray 25.5 × 55.5-cm cards (35% reflectance). Each card has a small peephole (4 mm in diameter) in the center to allow the experimenter to observe the child’s looking behavior. Each card also features a 12 × 12-cm square-wave grating (black and white stripes, at approximately 95% contrast) on one side of the central peephole. Gratings range from 0.32 to 38.0 cycles/cm in approximately half-octave steps. The space-averaged luminance of each grating is equal to the gray background of each card.

Patients sat on an adult’s lap facing an observer holding the card. The cards were presented by the observer to the child from a distance of 38 cm. All children were examined at a distance of 38 cm regardless of age due to the attentional, neurological, and eye motility issues (such as nystagmus) of the children evaluated in this study. At the start of each acuity measurement, the observer was always blind to the left–right location of the grating on the card. The experimenter then attracted the child’s attention to the card and observed the direction of her gaze through the peephole. The observer’s task in each trial was to make a forced-choice guess about the location of the grating based on the child’s behavior (primarily the direction of the gaze). An assistant, behind the child, who could see the grating’s location, recorded the observer’s guess on each trial and gave feedback.

A one-down, one-up staircase procedure was used during the test. Firstly, the card with the 0.23-cycles/cm spatial frequency stimulus (the first card in the set) was presented to account for the possibility that children with very low acuity could be present in the sample. A card with a half-octave higher spatial frequency was selected every time the observer made a correct guess on the grating position, but one with a half-octave lower spatial frequency every time the observer made an incorrect guess. The staircase was completed after a minimum of three reversals depending on the experimenter’s confidence about his judgment of the children’s responses. A threshold for VA was calculated as the geometric mean of the spatial frequencies of the gratings in the final three reversals.

The VA thresholds were converted to logarithm of the minimum angle of resolution (logMAR) based on the distance of 38 cm common across all participants. If participants had a prescription for refractive correction, and used well-adapted spectacles, they performed the test wearing them. Children born prematurely had their age corrected (from postnatal to postterm), and their VA scores were compared to postterm age norms assuming that there were no differences in the VA between terms and preterms (30–34).

Children were subjected to two sets of brief tasks of functional vision assessment—15 items related to the visual function of the Bayley Scales of Infant and Toddler Development, third edition (Bayley-III, 2006), were selected (nine in the cognitive set and six in the motor set)—on the same occasion their VA was being tested, with the aim of examining whether there was any relationship with the measured VA. A complete application of the developmental outcomes for the patients in the cohort (at a younger age) has been published previously (35).

The VA values were compared with the normative values established by Salomão and Ventura (22) using the same tolerance intervals. The patients were categorized according to their VA scores falling above, marginally below, or below normative values. The effects of both the patient’s age and the presence of any kind of retinal damage on VA were investigated by estimating the coefficients in a linear regression model. Confidence intervals for the coefficients and the likelihood ratio (LR) statistic (36) were calculated. The relationship between acuity and functional visual outcomes was evaluated using a logistic regression statistical model. We established the criterion of having successfully completed at least 2/3 of the investigated VA tasks as the binary outcome for the model, which was related to the VA value (in logMAR) as the independent variable. Any associations were considered significant if the calculated LR statistic value was above the 0.05 significance level. The statistical routines from the statsmodels library (37) were used for all statistical procedures.

## Results

We evaluated 45 children, 41 of whom met our inclusion criteria (20 boys). The age range was 21–34 months. Diagnosis of CZS based on a positive RT-PCR test result from either the mother and/or the child was available in 18 cases. For the remaining 23 cases, the diagnosis was based on the pregnant mother showing ZIKV symptoms and the child presenting CZS outcomes, such as microcephaly. Within the group of children clinically diagnosed with ZIKV infection but without positive laboratory confirmation, either the child or the mother had a negative result in 14 cases at the time of testing (which does not rule out viremia at an earlier stage relative to the testing occasion), while nine cases were untested. There were 26 co-occurrences of microcephaly and fundoscopic alterations and a single case of ZIKV-infected child with fundoscopic alterations in the absence of microcephaly. In all cases included based on clinical criteria, the mothers tested negative for exposure to TORCH (*Toxoplasma gondii*, other agents, rubella, cytomegalovirus, and herpes simplex virus) agents.

**Figure 1:**
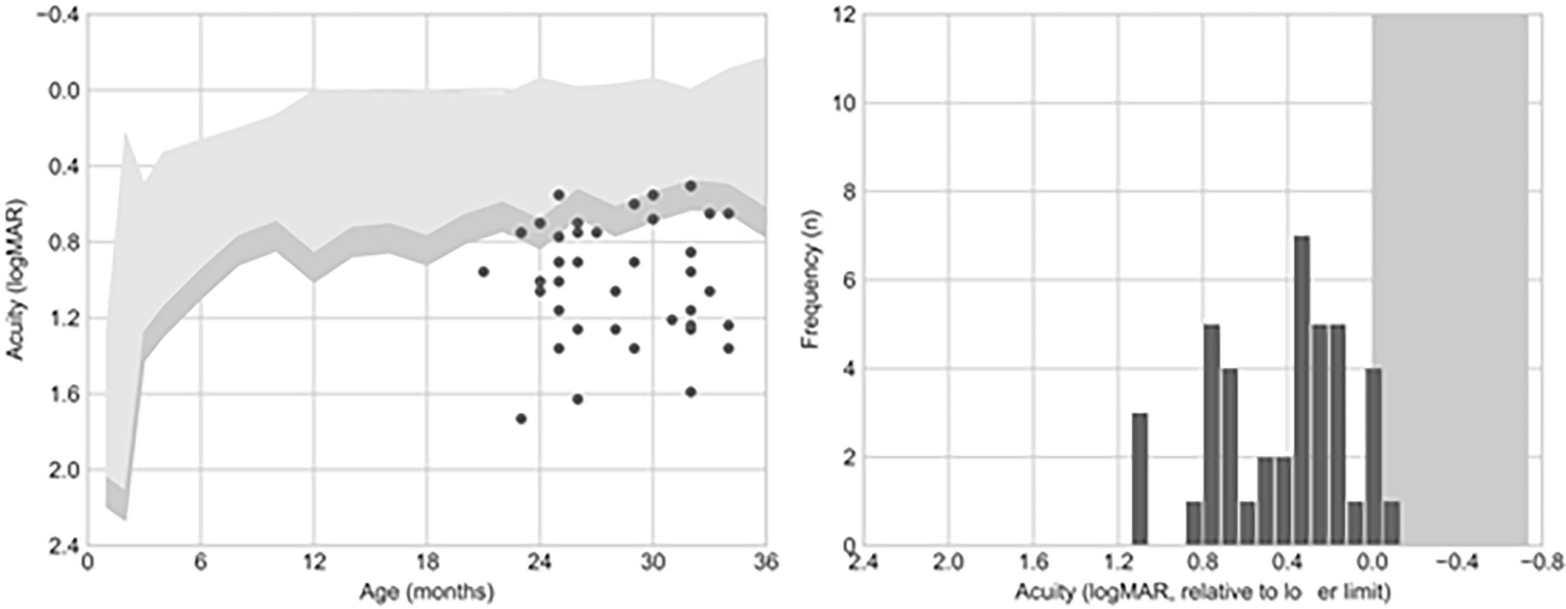
Visual acuity (VA) outcomes. The *left panel* shows the comparison of VA to the normative values published by Salomão and Ventura (1995). The *right panel* shows acuity as an offset from the mean acuity for the patient’s age. Six out of 41 (15%) patients presented an acuity value falling below this criterion.

The patients in the sample had a mean VA of 1.0 logMAR (SD = 0.3, range = 1.73–0.5). Most of the children (39/41, 95%) had VA scores below the normative values for their age. In 10 cases, VA was only marginally below the normative values (VA within 0.15 logMAR from the lower normative limit, equivalent to a single spatial frequency step in the card set); for the remaining 29 cases, VA was more than 0.15 logMAR below the lower limit (**Figure 1**).

VA measurements within 0.15 logMAR of the lower normative limit require repeated testing and/or additional clinical information collected *via* other techniques before diagnosing VA deficits (Stereo-Optical Co., 2005). Among the 10 children with VA only marginally below the normative values, 7 (70%) presented retinal damage. In the group of children with VA more than 0.15 logMAR below the inferior limit, 21 (72%) presented retinal damage. The mean and range of the VA values, and their association with retinal damage, using this classification are summarized in **Table 1**.

**Table 1:** Visual acuity (VA) by classification.

Classification	*N*	Minimum acuity (logMAR)	Maximum acuity (logMAR)	Eye damage (*N*)
Above inferior limit	2	0.6006	0.5527	1
Marginally below inferior limit (<0.15 logMAR)	10	0.9574	0.5048	7
Below inferior limit (>0.15 logMAR)	29	1.7316	0.7010	23

logMAR, logarithm of the minimum angle of resolution.

**Figure 2:**
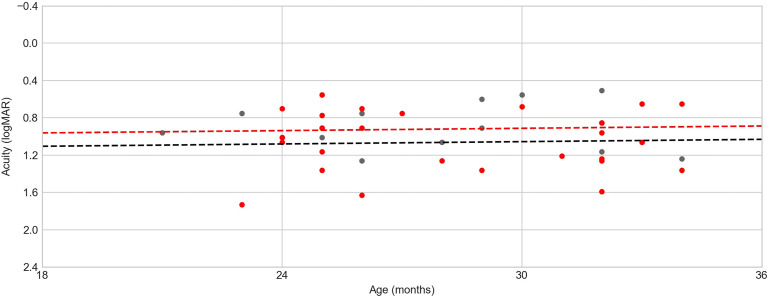
Age (in months) × visual acuity (VA). *Red dots* denote children with some kind of fundoscopic alteration, *black dots* indicate children without fundoscopic alterations, and *dotted lines* represent the age × acuity regression model for each group (*red line* for the group of children with fundoscopic alterations and *black line* for the group of children without fundoscopic alterations). There is no tendency because of the homogeneity in the children’s ages.

A multiple linear regression model that included age (in months) and the presence of any fundoscopic alterations as independent variables predicting VA (in logMAR) showed no significant effect for either variable (age = −0.0278 to 0.0237 logMAR/month, retinal damage = −0.0564 to 0.03424 logMAR change, LR = 2.08, *p* = 0.354). In the age range tested (21–34 months), only a very modest increase in VA was expected (0.2 logMAR in the average of the normative values); consequently, it was not possible to evaluate the problems in the development of VA in this sample (**Figure 2**). Since all children were tested binocularly, the absence of a relationship between eye damage and acuity could be due to compensation by an unaffected eye.

The functional vision examination results are summarized in **Table 2**. Overall, most patients were able to complete simpler tasks (e.g., paying attention to an object and reacting to the examiner’s face occlusion), but only a few completed more complex tasks (e.g., persistent reaching, preference for a novel object).

**Table 2:** Functional vision evaluation.

Task domain	Task	No. of patients	Proportion (%)
Cognitive domain	Item 3—Pays attention to object (3s)	32	78.04
	Item 8—Pays attention to object (5s)	32	78.04
	Item 9—Reacts to face occlusion	35	85.36
	Item 11—Shows visual preference	28	68.29
	Item 12—Habituates to object	18	43.90
	Item 13—Prefers new object	9	21.95
	Item 15—Prefers new object figure	6	14.63
	Item 17—Takes object to mouth	23	56.09
	Item 21—Persistent reaching	6	14.63
Motor domain	Item 2—Eyes follow moving person	31	75.60
	Item 3—Eyes follow plastic ring (horizontal)	22	53.65
	Item 4—Eyes follow plastic ring (vertical)	23	56.09
	Item 7—Eyes follow plastic ring (circular)	18	43.90
	Item 8—Head follows plastic ring	19	46.34
	Item 9—Eyes follow moving ball	14	34.14

To relate functional vision evaluation to acuity, we identified which patients performed at least two-thirds of the tasks successfully. Meeting this criterion was taken as a dependent binary variable in the logistic regression model, with the measured VA in logMAR as its independent variable. No relationship could be established between VA and the cognitive domain tasks (acuity regression coefficient = −0.87 to 0.43 log odds change, LR = 0.475, *p* = 0.4905), but there was a statistically significant relationship between VA and the motor domain tasks (acuity regression coefficient = −1.00 to −0.02 log odds change, LR = 4.109, *p* = 0.0426). Moreover, it was interesting to observe that only four children did not complete at least one task in both sets of tasks (cognitive or motor), all of them part of the most impaired group (below normative values). Most of the children (37) performed at least one task in the cognitive set, but 14 children did not perform even one task in the motor set. These results may imply that the visual deficits in these children have impaired their motor skills even when there is less damage to their cognitive skills. Furthermore, the children above the lower VA limit per age showed a better mean performance in the cognitive (67%) and motor (66%) sets than the children whose VA fell below the lower normative limit. Those with scores that were marginally below the VA lower limit showed 49% and 38% mean performance in the cognitive and the motor set, respectively, while those with VA below the lower limit had 48% and 44% mean performance in the cognitive and the motor set, respectively.

## Discussion

The present study adds to the literature in this area and furthers our research team’s efforts to characterize the VA and VA development losses in children exposed to ZIKV infection during pregnancy, as we have done in previous studies (17, 18) examining a cohort from Jundiaí, São Paulo, Brazil’s southeast. In this work, we evaluated a distinct population in Brazil’s southeast, in the city of Rio de Janeiro (Rio de Janeiro), that included patients who were older (previous studies included patients ranging from 4 to 13 months of age (2, 12, 13), while the patients in the present study had a median age of 24months), were more severely affected by CZS, had different epidemiological profiles, and were enrolled in a cohort that followed a different design.

Compared to the Jundiaí cohort, which documented only a few cases of microcephaly, the Rio de Janeiro cohort tells a very different story, presenting a much larger number of children with CZS in which fundoscopic alterations, VA losses, and microcephaly are likely more intertwined than in the Jundiaí cohort. In the Rio de Janeiro cohort, only one patient did not have microcephaly (but had fundoscopic damage). Eight of the 41 children with microcephaly did not have ophthalmological anomalies, while 7 of the 41 children showed VA within or marginally below the normative values. In other words, in this cohort, all children with VA loss also had microcephaly and/or ophthalmological damage, which made it more difficult to determine whether the VA losses in these children were due to neurological alterations, fundoscopic alterations, or both.

The children sampled for the Rio de Janeiro cohort were from a larger study, which showed that, beyond the fundoscopic alterations (mainly damages in the retina and optic nerve) (15), these children also had ocular motility damage (11), which is in agreement with the high degree of VA losses we found in this sample.

The children evaluated in the Rio de Janeiro and Jundiaí cohorts were similar in size, with respectively 40 and 23 children in each one, but their profiles were significantly different. All subjects in the Rio de Janeiro cohort had microcephaly and/or ophthalmological impairment, against only 16% (4/24) of children with microcephaly in the Jundiaí cohort. Most of the children (84%) in the Rio de Janeiro cohort had VA below or marginally below the normal, against 21% of the children with subnormal VA in the Jundiaí cohort. The results in our samples appear to be representative of the cohorts as a whole, given that, in the Jundiaí cohort, from 695 pregnant mothers initially accompanied, only 53 (7.6%) were confirmed to have ZIKV infection, of whom only 35% of the liveborn children had confirmed ZIKV infection and only 4.5% had microcephaly (38, 39). On the other hand, in the Rio de Janeiro cohort, from 224 infants followed since birth, 156 (70%) had confirmed ZIKV infection, of whom 62 (40%) had microcephaly (11, 15, 35). In other words, despite their different sizes (Jundiaí being a much larger cohort than Rio de Janeiro), the children included in the Rio de Janeiro presented 10 times more confirmed ZIKV infection and, in these infected children, a 10 times greater chance of presenting microcephaly.

Although these differences may be partly due to the different selection criteria used by the IFF Fiocruz research team (while the Rio de Janeiro cohort followed only symptomatic pregnant mothers or children with suspected ZIKV infection, the Jundiaí cohort followed pregnant women independently of the presence of ZIKV infection symptoms), this alone does not explain the different numbers of microcephaly and fundus alterations between the two samples, nor the differences in the incidences of ZIKV infection and microcephaly between them. These differences could be due to differences in the virus strains (40, 41) present in the two states. They could also be caused by differences in the immunological resistance between the two populations, due to previous exposure to other viral agents, nutritional profiles, or genetic differences (26).

Considering that most of the children (95%) evaluated here had VA below the lower normative values per age, it is important to note that 26% were only marginally below that limit. This, coupled with the fact that most children (92%) performed at least one task in the visual function set, suggests that some of them, although heavily visually impaired, may benefit from a visual rehabilitation program, which might contribute to mitigating some of the visual damage suffered.

Only binocular evaluations were performed due to time constraints, which made it difficult to directly relate the VA outcomes with retinal damage since visual losses in the affected eye in monocular lesions might be compensated by the unaffected eye. Additionally, the measured VA varied widely in the investigated sample, and attentional, neurological, and eye motility issues (such as nystagmus) may have contributed to this variability. Several precautions were taken to minimize such factors (displaying the cards vertically for children with horizontal nystagmus and testing at a 38-cm distance for all children), but these potential extraneous influences must be considered.

Most of the children (87%) assessed in our study were older than 24 months at the time of testing. No studies dedicated to CZS have reported VA measurements for this age range yet. The VA assessments at this age range have fewer developmental sources of variability and might offer a VA measurement that best captures the visual function of these patients at school age and later in adulthood. However, since this age range has a very slight increase in VA development, it was not possible to evaluate the development of VA in this sample.

## Limitations

A limitation of the present study is the fact that only 18 cases of ZIKV infection were confirmed by PCR; in fact, 14 cases, between mother and children, tested negative. Nevertheless, to minimize this limitation, all of the cases included in our study were clinically confirmed and in line with what was expected of children affected with CZS. Moreover, for all cases included based solely on clinical criteria, all mothers tested negative for TORCH agents.

## Data availability statement

The raw data supporting the conclusions of this article will be made available by the authors, without undue reservation.

## Ethics statement

The studies involving human participants were reviewed and approved by the Ethics Committee for Human Research of the University of São Paulo’s Institute of Psychology and Ethics Committee for Human Research of the Instituto Fernandes Figueira (IFF)—Fiocruz. Written informed consent to participate in this study was provided by the participants’ legal guardian/next of kin. Written informed consent was obtained from the minor(s)’ legal guardian/next of kin for the publication of any potentially identifiable images or data included in this article.

## Author contributions

LCPB and DL collected and analyzed the data and wrote the manuscript. LS collected additional data and helped in the writing of the manuscript. HT and SD helped collect the data. AZ and MM provided the patients examined and the locality for examination and supplied additional data. MC helped collect and analyze the data and also revised the manuscript. Senior author DV supervised all work and revised the manuscript. All authors contributed to the article and approved the submitted version.

## Funding

This work was supported by grants to DFV from Sao Paulo State Research Foundation (FAPESP) Thematic Projects #2014/26818-2 and #2022/00191-0 and from Coordination for the Improvement of Higher Level Education Personnel (CAPES) PróAmazonia 2013/3263. LCPB (no. 2016/14793-0), DL (no. 2016/24631-8), LS (no. 2017/16948-4), and HT (no. 2016/21573-7) had MSc student fellowships from FAPESP. DL had a CAPES doctoral student fellowship. LCPB had a doctoral student fellowship from FAPESP (n. 2019/18487-0). DFV (314630/2020-1) and MC (306020/2020-3) are National Council for Scientific and Technological Development (CNPq) Productivity Research Fellows. Additional grants were: CNPq 441098/2016-9 and 305090/2016-0; FaperjE_18/2015TXB; Wellcome Trust and the United Kingdom’s Department for International Development (205377/Z/16/Z); European Union’s Horizon 2020 Research and Innovation Programme under Zika-PLAN grant agreement no. 734584. The sponsors or funding organizations had no role in the design or conduct of this research.

## Conflict of interest

The authors declare that the research was conducted in the absence of any commercial or financial relationships that could be construed as a potential conflict of interest.

## Publisher’s note

All claims expressed in this article are solely those of the authors and do not necessarily represent those of their affiliated organizations, or those of the publisher, the editors and the reviewers. Any product that may be evaluated in this article, or claim that may be made by its manufacturer, is not guaranteed or endorsed by the publisher.
